# Genetic Signatures of Contrasted Outbreak Histories of “*Candidatus* Liberibacter asiaticus”, the Bacterium That Causes Citrus Huanglongbing, in Three Outermost Regions of the European Union

**DOI:** 10.1111/eva.70053

**Published:** 2024-12-16

**Authors:** Olivier Pruvost, Karine Boyer, Frédéric Labbé, Marine Weishaar, Anaïs Vynisale, Claire Melot, Cécile Hoareau, Gilles Cellier, Virginie Ravigné

**Affiliations:** ^1^ CIRAD UMR PVBMT Saint Pierre France; ^2^ Plant Health Laboratory, Anses Saint Pierre France; ^3^ CIRAD UMR PHIM Montpellier France

**Keywords:** bacterial crop disease, miniature inverted‐repeat transposable elements, molecular epidemiology, population structure, prophage, tandem repeats

## Abstract

In an era of trade globalization and climate change, crop pathogens and pests are a genuine threat to food security. The detailed characterization of emerging pathogen populations is a prerequisite for managing invasive species pathways and designing sustainable disease control strategies. Huanglongbing is the disease that causes the most damage to citrus, a crop that ranks #1 worldwide in terms of fruit production. Huanglongbing can be caused by three species of the phloem‐limited alpha‐proteobacterium, “*Candidatus* Liberibacter,” which are transmitted by psyllids. Two of these bacteria are of highest concern, “*Ca*. Liberibacter asiaticus” and “*Ca*. Liberibacter africanus,” and have distinct thermal optima. These pathogens are unculturable, which complicates their high‐throughput genetic characterization. In the present study, we used several genotyping techniques and an extensive sample collection to characterize *Ca*. Liberibacter populations associated with the emergence of huanglongbing in three French outermost regions of the European Union (Guadeloupe, Martinique and Réunion). The outbreaks were primarily caused by “*Ca*. Liberibacter asiaticus,” as “*Ca*. Liberibacter africanus” was only found at a single location in Réunion. We emphasize the low diversity and high genetic relatedness between samples from Guadeloupe and Martinique, which suggests the putative movement of the pathogen between the two islands and/or the independent introduction of closely related strains. These samples were markedly different from the samples from Réunion, where the higher genetic diversity revealed by tandem‐repeat markers suggests that the disease was probably overlooked for years before being officially identified in 2015. We show that “*Ca*. Liberibacter asiaticus” occurs from sea level to an altitude of 950 m above sea level and lacks spatial structure. This suggests the pathogen's medium‐ to long‐distance movement. We also suggest that backyard trees acted as relays for disease spread. We discuss the implications of population biology data for surveillance and management of this threatful disease.

## 1 Introduction

The impact of globalization, that is, the increase in long‐distance transport of goods and humans, not to mention climate change, exacerbates the emergence of pests and pathogens (Gardy and Loman 2018; Garrett et al. 2022; Paini et al. 2016; Santini et al. 2018). Major crop pests and pathogens threaten agricultural production and global food security (Perrings 2016; Savary et al. 2019). Population biology of pathogens, a discipline that bridges epidemiology and population genetics, has succeeded in refining our knowledge of crop pathogen emergence (Milgroom 2015). Some of the data and analyses generated have effectively helped improve disease surveillance and management, including breeding for resistance and resistance genes or antimicrobials deployment (Saubin et al. 2023; Zhan et al. 2015). The progress in the field of pathogen population biology has made it possible to consider important issues to help us understand and manage pathogen emergence, such as (i) the number of introduction events associated with outbreaks (a parameter that can greatly influence adaptation at landscape scales); (ii) the estimation of dispersal parameters in conditions of heterogeneous inoculum at local to continental spatial scales; (iii) the identification of sources of primary inoculum and its genetic relatedness to previous known strains, dissemination pathways or invasion routes; and (iv) characterization of adaptive traits linked to emergence (e.g., changes in aggressiveness, host/tissue specialization, and resistance to antimicrobials), or ecological changes with an impact on pathogen success (e.g., climate change, land use, host demography, or behavior) (Milgroom 2015; Rasmussen and Grunwald 2021; Woolhouse 2002).

Citrus is widely grown in many regions and generates more economic value than any other fruit crop in the world (Talon, Caruso, and Gmitter Jr. 2020). Citrus industries suffer from several major biotic stresses, including three severe bacterial diseases: Asiatic citrus canker, citrus variegated chlorosis, and citrus huanglongbing (HLB; synonym citrus greening), which is regarded as the most destructive one (Gabriel et al. 2020). Aetiology of HLB was only firmly established in the 1980s (Garnier, Danel, and Bove 1984a, 1984b). Three non‐culturable species of the alpha‐proteobacterium “*Candidatus* Liberibacter” were reported to cause HLB (Jagoueix, Bové, and Garnier 1994; Teixeira et al. 2005). In terms of economic impact, the most important citrus bacterial pathogen worldwide is undoubtedly “*Candidatus* Liberibacter asiaticus” (CLas) (Bové 2006; Gottwald, Graça, and Bassanezi 2007). The two other HLB‐causing species are “*Ca*. Liberibacter africanus” (CLaf) and “*Ca*. Liberibacter americanus” (CLam). They have a more restricted geographic distribution and are both sensitive to high temperatures (DaGraca et al. 2022; Lopes et al. 2009). HLB‐causing *Ca*. Liberibacter cells are directly injected into citrus phloem cells by two species of psyllid vectors: the Asian citrus psyllid (ACP; *Diaphorina citri* ) and the African citrus psyllid (AfCP; *Trioza erytreae*). These pathogens are also graft transmissible (Bové 2006). HLB has an impact on citrus fruit yield and quality, and causes massive loss of root mass and tree death. A detailed description of HLB symptoms is available elsewhere (Bové 2006; Gottwald, Graça, and Bassanezi 2007). Similar to other insect‐transmitted bacteria, the genome size of HLB‐causing *Ca*. Liberibacter was drastically reduced during its evolution and it remains routinely unculturable. These features complicate the study of its metabolism, interactions with insect or plant hosts, and evolution (Huang et al. 2020; Wang 2019). Early disease diagnostics is challenging because the latent infection period is exceptionally long and the sensitivity of molecular detection assays is suboptimal. However, real‐time PCR has partially addressed this issue (Gottwald 2010).

‘*Candidatus* Liberibacter asiaticus’ probably originated from Asia and is now found in several major citrus‐producing basins (Asia and the Americas, with a limited presence in Africa). It is not yet established in two main production areas, the Mediterranean Basin and Australia (Bové 2006; DaGraca et al. 2022; Gottwald 2010; Huang et al. 2020). In mainland European Union (EU), HLB is a major concern because both of its insect vectors, ACP and AfCP, have been detected in Cyprus (EPPO Global Database; https://gd.eppo.int/reporting/article‐7660), and in Spain and Portugal (Arenas‐Arenas et al. 2019; Siverio et al. 2017), respectively.The *Ca*. Liberibacter species that cause HLB are currently listed among the EU's top 20 priority pests (EU 2019/1702). To date, HLB has been reported in three outermost regions of the EU: Réunion, Guadeloupe, and Martinique. This may represent a source of *Ca*. Liberibacter for mainland EU because of the political and economic links with these regions (Aubert, Bové, and Etienne 1980; Cellier et al. 2014).

Symptoms corresponding to HLB were first reported in 1968 in Réunion (Aubert, Bové, and Etienne 1980). The causal agents were subsequently identified as CLas and CLaf (Garnier et al. 1996). At the time, these species were reported in the lowlands and highlands, respectively, with an intermediate altitudinal range where both pathogens occurred, as co‐infections in some cases.

The two psyllid species involved in HLB transmission, ACP and AfCP, were found to be associated with this outbreak (Aubert, Bové, and Etienne 1980; Bové 2006). After a long and massive control program involving the removal of diseased trees, chemical control (targeting bacteria and insect vectors) and biological control of both psyllid species (using the parasitoid species, *Tamarixia radiata* and *T. dryi*), disease incidence decreased to very low levels in the 1990s (Aubert 2008; Aubert et al. 1996; Bové 2006). After the highly effective biological control of vectors in Réunion, citrus replanting was undertaken. However, there was no active surveillance of CLas and CLaf, partly because of the lack of sensitive laboratory detection techniques at that time (Manjunath et al. 2008). In 2015, HLB‐like symptoms related to CLas were again reported from the historical area of citrus cultivation (southern part of the island). Since then, HLB has been found in most areas where citrus are cultivated. The two insect vector species are still present in Réunion, although AfCP has a much more limited distribution (Reynaud et al. 2022). In the two outermost Caribbean regions, Guadeloupe and Martinique, ACP was first detected in 1998 and 2012, respectively, whereas CLas was first detected in 2012 and 2013, respectively (Cellier et al. 2014). The origin of the introductions of CLas in these three EU outermost regions remains unknown.

An increasing number of complete CLas genomes have been made available since 2009. The epidemiology of bacterial pathogens has generally benefitted from the rapid development of high‐throughput whole‐genome sequencing (WGS) over the past decade (Gardy and Loman 2018; Stam et al. 2021). However, the unculturable status of HLB‐causing bacteria remains an obstacle to its wider implementation. The genotyping data used in molecular epidemiology studies published to date rely primarily on molecular markers, for example, tandem repeats (TRs) used in a multilocus genotyping format (MLVA) and/or prophages (Dominguez‐Mirazo, Jin, and Weitz 2019; Zheng et al. 2016, 2018), which may host miniature inverted‐repeat transposable elements (MITEs) (Cui et al. 2022; DaSilva et al. 2019; Das et al. 2021; Islam et al. 2012; Katoh et al. 2011; Wang et al. 2013; Zheng et al. 2021; Zheng et al. 2016). Based on these genotyping techniques, studies conducted in China and India (where HLB became established more than half a century ago) indicate that several distinct, geographically structured populations occur in different provinces/states (Das et al. 2021; Gao et al. 2022; Ghosh et al. 2015; Zheng et al. 2021). Although counter examples do exist (in Brazil) (DaSilva et al. 2019), signatures of putative multiple introductions have also been recorded at the scale of a single province/state, even in places where the pathogen's establishment is fairly recent (e.g., California, United States) (Dai et al. 2019). Interestingly, these studies also indicated that abiotic factors (e.g., altitude) and human activities (e.g., transport of propagative plant material) have a putative influence on the observed structure. This confirms the value of genotyping for deciphering the occurrence of multiple independent introduction events (Armstrong et al. 2022; Dai et al. 2019; Das et al. 2021; Fu et al. 2020; Zheng et al. 2021).

Here, we analyzed the population structure of CLas in each of the three EU outermost regions where HLB outbreaks have been reported. We considered their genetic relatedness and the plausibility that genetically remote pathogen populations may have been independently introduced in these territories on several occasions. For this, we used MLVA, prophage, and MITE typing. We built on the seven‐loci scheme, previously developed by Islam et al. (2012), and designed a new MLVA scheme targeting 12 TR loci (10 microsatellites and 2 minisatellites) for improved resolution. The present study is the first extensive molecular characterization of a comprehensive citrus sample collection originating from these EU outermost regions.

## 2 Materials and Methods

### 2.1 Biological Material and Its HLB Status

**FIGURE 1:**
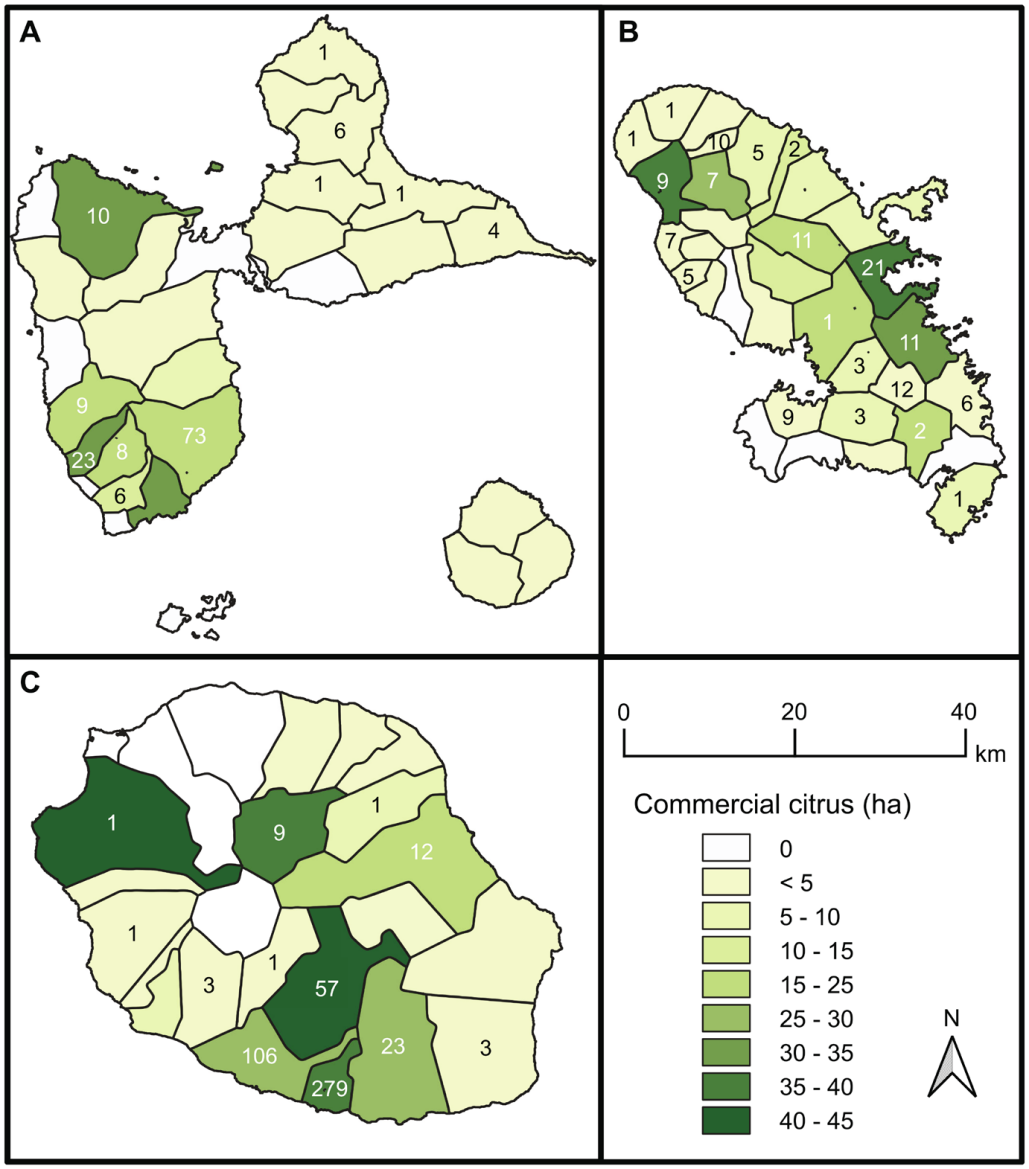
Geographical distribution of HLB‐positive (as determined by real‐time PCR; Cq ≤ 30) gDNAs in (A) Guadeloupe, (B) Martinique, and (C) Réunion. All these gDNAs were genotyped using tandem‐repeat markers (MLVA‐12). Shades of green indicate the commercial citrus surface area in each locality. In addition to the samples counted on the map, 11 samples were taken in municipalities with a very low density of commercial citrus production (not shown on the map in compliance with statistical confidentiality).

Genomic DNAs (gDNA) were purified from citrus leaf samples originating from all areas of citrus cultivation in Guadeloupe, Martinique, and Réunion. The geographical distribution of HLB‐positive samples is shown in Figure 1. Three sources of HLB‐positive gDNA were used in this study:

- Set (i): A total of 148, 129, and 129 HLB‐positive gDNAs transmitted by diagnostic laboratories from samples collected in Guadeloupe (2013–2020), Martinique (2013–2020), and Réunion (2015–2022), respectively. Each gDNA was purified from pooled leaves (1.0 g of midrib) sampled from several symptomatic trees from a same citrus block. HLB status of these samples was determined by diagnostic laboratories using the official French analysis endpoint PCR protocol (ANSES/LSV/MA 033—Version 1b—December 2014; https://www.anses.fr/fr/system/files/ANSES_LSV_MOA033_V1b.pdf). The associated sampling covered all areas of commercial citrus cultivation of each island.
- Set (ii): 313 HLB‐positive (real‐time PCR Cq ≤ 30) gDNAs derived from 1692 leaf samples collected from single trees in commercial groves by the CIRAD in Réunion between 2018 and 2022 (one to five symptomatic trees sampled per block). To test intra‐host potential variability, five leaves were collected per tree by the CIRAD Réunion from 2018 to 2020 (i.e., a total of 291 trees). This allowed to collect samples from additional commercial blocks and whenever possible (i.e., when trees had not been pulled out) to individually resample trees for which gDNAs produced by diagnostic laboratories yielded MLVA multiallelic states at several TR loci (i.e., because of the tree pooling strategy used).
- Set (iii): 48 HLB‐positive (real‐time PCR Cq ≤ 30) gDNAs derived from 238 leaf samples collected from backyards in all four Réunion districts (elevation ranging from 49 to 984 m above sea level (masl)) by the CIRAD between 2021 and 2022. Each sample collected by the CIRAD consisted of five symptomatic leaves from one tree. HLB status of these samples was determined as described in the following.

We determined the HLB status of all samples from purified gDNAs according to the official French analysis real‐time PCR protocol (ANSES/LSV/MA 063—Version 2—October 2021; https://www.anses.fr/fr/system/files/ANSES_LSV_MA063_V2.pdf). Briefly, the DNeasy Plant mini kit (Qiagen, Courtaboeuf, France) was used according to the manufacturer's recommendations for plant tissue. The gDNA concentration and quality were assessed using the NanoDrop ND8000 (Thermofisher, Les Ulis, France). The gDNAs were assayed as duplicates for the presence of CLas with the real‐time PCR assay, developed by Li, Hartung, and Levy (2006), and using the GoTaq Probe qPCR master mix as recommended by the manufacturer (Promega, Charbonnières‐les‐Bains, France), the StepOnePlus cycler, and the Design and analysis software, v.2.5 (Applied Biosystems, Courtaboeuf, France). As CLaf is a temperature‐sensitive species (DaGraca et al. 2022) and was found in previous decades in Réunion (Garnier et al. 1996), all (*n* = 115) samples collected at an elevation ≥ 800 masl were also submitted to the CLaf real‐time PCR assay (Li, Hartung, and Levy 2006). This elevation corresponds to the altitude at which AfCP was mostly detected in Réunion during the 1970s–1980s outbreak (Aubert 1987). A subset of 40 samples from Réunion that was positive for both CLaf and CLas, using the Li, Hartung, and Levy (2006) real‐time PCR assay (because of assay specificity issues; see Morán et al. 2021 and Roberts et al. 2017), was also assayed by the French Anses reference laboratory with recently improved primers, which target the same region as Li et al. (Osman et al. 2023). Primers recommended for CLaf and CLas were used separately. Briefly, 1 μL of each sample was analyzed in a final volume of 12 μL, containing 0.08 μM of probe and 0.40 μM of each primer, along with 2X GoTaq Probe qPCR master mix (Promega, Charbonnières‐les‐Bains, France), under the following conditions: 95°C for 10 min, followed by cycles with denaturation at 95°C for 15 s and annealing–elongation at 60°C for 60 s, as recommended (Osman et al. 2023).

### 2.2 Identification at the Species Level Using Multilocus Sequence Analysis (MLSA)

Consistent with previous studies (Morán et al. 2021; Roberts et al. 2017), the real‐time PCR assay developed by Li, Hartung, and Levy (2006) yielded positive results for both CLas and CLaf for all markedly positive samples. Although the species status could be predicted as the primer/probe system with the lowest Cq value, the Cq difference among both systems was repeatedly found < 1, yielding putatively ambiguous results. As expected, this was also the case for the more recently developed primers (Osman et al. 2023). Consequently, we adapted the MLSA scheme originally developed for *Ca*. Liberibacter solanacearum (Haapalainen et al. 2018) to HLB‐causing *Ca*. Liberibacter for identification at the species level. Seven gene portions, *adk*, *atpA*, *fbpA*, *ftsZ*, *glyA*, *groEL*, and *gyrB*, were targeted. Primers for PCR amplification were designed from the CLas psy62 (Duan et al. 2009) and CLaf PTSAPSY (Lin et al. 2015) genomes (Table S1) using the Geneious R10.2.6 software (Biomatters Ltd., Auckland, New Zealand) (Duan et al. 2009; Lin et al. 2015). PCR was performed using the BD advantage 2 polymerase mix kit (Takara Bio, Saint‐Germain‐en‐Laye, France). Briefly, 2 μL of 10‐fold‐diluted DNA was used as a template in mixes containing 5 μL of 10× Advantage 2 PCR Buffer SA 10×, 0.2 mM of dNTPs mix, 0.2 μM of each primer (Table S2), and 1 μL of Advantage 2 polymerase mix and RNase‐free water to yield a final volume of 50 μL. PCR amplifications were performed in a Veriti thermocycler (Applied Biosystems, Courtaboeuf, France) under the following conditions: 3 min at 95°C (HotStart activation and DNA initial denaturation), 40 cycles with denaturation at 95°C for 15 s (primer annealing temperatures: Table S2) amd for 30 s, extension at 68°C for 1 min, and then a final extension at 68°C for 7 min. Amplicons were sequenced by Genewiz (Takeley, United Kingdom) using the same primers as for PCR. Preliminary analyses (data not shown) suggested that only samples for which real‐time PCR Cq values were < 30 readily produced amplicons with the MLSA primers. Therefore, all markedly (Cq ≤ 30) CLas‐positive samples collected at high elevation (≥ 800 masl; *n* = 3) and the putative CLaf samples (*n* = 4) (i.e., markedly positive samples with slightly delayed Cq values recorded for the CLas assay compared to the CLaf assay) were further analyzed using MLSA. They were compared to (i) representatives of all species of *Liberibacter* and *Ca*. Liberibacter for which genomic resources are publicly available (Table S1) and (ii) a subset of 11 reference samples originating from the French West Indies (Guadeloupe *n* = 3 and Martinique *n* = 8) and 11 from Réunion (including samples from the first official detection in 2015) collected from the main areas of commercial cultivation (Table S1).

During MLSA, two regions in *atpA* and *ftsZ* that were potentially derived from recombination (i.e., regions identified as recombinant by at least four detection methods using RDP v.5.34, Martin et al. 2015) in the concatenated dataset were filtered prior to tree construction. The most appropriate evolutionary model (GTR + G) was selected, based on a jModelTest v.2.1.10 analysis (Posada 2008). Maximum‐likelihood (ML) methods were used to infer phylogenetic relationships among *Liberibacter* and *Ca*. Liberibacter species using PhyML v.3.3 (Guindon et al. 2010). The produced tree was rooted on *Liberibacter crescens*, that is, the most differentiated species according to the distance matrix.

### 2.3 Microsatellite Marker Development

TR loci typically follow a generalized stepwise mutation model and thus suffer by nature of size homoplasy (i.e., identical alleles in state but not by descent) (Ellegren 2004; Vogler et al. 2006). This limitation can be minimized by using genotyping schemes that target a sufficiently large number of loci (Estoup, Jarne, and Cornuet 2002; Reyes, Chan, and Tanaka 2012). Therefore, additional TR loci were mined to complement those developed previously (*n* = 7) (Islam et al. 2012). The psy62 CLas genome sequence (Duan et al. 2009) was chosen as our reference (Table S1) and screened using Tandem Repeats Finder in the dedicated online resource (https://tandem.bu.edu/trf/trf.html; Benson 1999) and the Phobos v.3.3.12 plug‐in of Geneious (parameters: 3 ≤ repeat size ≤ 100 bp; array size ≤ 500 bp; allowing for imperfect repeats). Flanking regions (500 bp up‐ and downstream of the TR loci) were used to define oligonucleotide primer pairs for PCR amplification with the Geneious software (Table S3). Compound TR loci and loci for which nucleotide identity of repeat units was < 70% were discarded. Selected TR loci and their flanking regions were submitted to blastN searches to check for presence and polymorphism in CLas strains. PCR optimization of universally‐present loci was performed using two reference CLas‐positive gDNAs from Réunion (V01 and V02). All selected loci were also blasted against CLaf genome sequence PTSAPSY (Genbank accession CP004021; Table S1), and none were present in this CLaf genome sequence.

### 2.4 CLas Genetic Polymorphism and Population Structure From TR Data

A total of 12 TR loci (MLVA‐12), including the seven previously published TR loci (MLVA‐7) (Islam et al. 2012), were used (Table 1). Preliminary analyses (data not shown) suggested that PCR amplification of microsatellite loci could not be reproducibly produced from weakly positive samples, that is, samples with a 30 < Cq < 36. The threshold officially used in France to qualify a HLB‐positive sample using real‐time PCR is Cq = 36. A total of 164, 113, and 513 samples (including 49 from backyards) from Guadeloupe, Martinique, and Réunion, respectively, with Cq values ≤ 30, were used for MLVA typing. Amplicons were produced from multiplex PCR, using the Type‐It Microsatellite PCR kit (Qiagen, Courtaboeuf, France). PCR mixes contained 7.5 μL of 2× Type‐It master mix (containing a hot‐start Taq DNA polymerase), 1.5 μL of 5× Q‐Solution, 0.3–0.7 μM of each primer (Table S3), 1 μL of DNA sample (diluted 10‐fold for samples with Cq < 25), and RNase‐free water to yield a final volume of 15 μL. PCR amplifications were performed in a Veriti thermocycler under the conditions recommended by the manufacturer and using a primer annealing temperature of 57°C. One microliter of 40‐ to a 100‐fold diluted amplicons were mixed with 10.5 μL of Hi‐Di formamide and 0.5 μL of a GeneScan 600 LIZ V2, as an internal size standard (Applied Biosystems, Courtaboeuf, France). Then, the mixture was denatured for 5 min at 95°C and placed on ice for at least 5 min. Capillary electrophoresis was performed in an ABI PRISM 3130xl genetic analyzer (Applied Biosystems, Courtaboeuf, France) using a performance‐optimized polymer, POP‐7, at 15,000 V at 60°C with an initial injection of 23 s. Reference samples V01 and V02 were used as controls in all experiments. As amplicon length estimates from capillary electrophoresis runs may vary depending on the equipment used, seven gDNA samples originating from Florida (FL47, FL117, and FL206) and China (CHN‐34, CHN‐39, CHN‐46, and CHN‐48) were obtained from USDA–ARS. They were genotyped and compared to the original data (Islam et al. 2012). Identical or very close amplicon sizes were obtained. gDNAs from set (i) sometimes yielded multiallelic profiles (i.e., as a consequence of the tree pooling strategy used by diagnostic laboratories). In such cases, genotyping was performed again. When multiallelic profiles were confirmed, corresponding tree bundles were resampled whenever possible, gDNA set (ii).

**TABLE 1:** TR markers used in the MLVA‐12 scheme on “*Candidatus* Liberibacter asiaticus” (CLas) samples from three EU outermost regions (*n* = 787).

TR name^a^	TR length (bp)	TR sequence	Position in psy62 genome	ORF	Range of TR numbers	Number of alleles (*H*_*T*_)^b^
T08	33	TCTGTTCTAACATCAGCTATATCTGCTTTAAGC	1,223,679 => 1,223,759	Hemolysin XhlA	2–5	4 (0.258)
T09	57	ATCAGGCAGGTTTTCTATTGCAATATCGATCTCACTAGCTTGATGGTTTGATTTCAA	1,009,710 => 1,009,874	Hypothetical protein	1–8	7 (0.736)
E	7	ACACAAG	354,492 => 354,528	Putative phage repressor protein	2–9	8 (0.737)
C	4	CAGT	537,729 => 537,760	Intergenic	5–10	6 (0.496)
T10	6	TTTTTA	405,894 => 405,905	Intergenic	4–7	3 (0.032)
T03	6	TTTAAT	360,544 => 360,573	Intergenic	5–7	3 (0.506)
F	8	TTTACATC	684,187 => 684,216	Intergenic	3–5	3 (0.048)
A	7	CAGAATA	255,593 => 255,648	Intergenic	4–25	18 (0.887)
T05	4	TTTG	655,274 => 655,332	Intergenic	8–19	11 (0.800)
B	5	TTAAT	535,168 => 535,195	Intergenic	5–7	3 (0.078)
G	8	TTGTTGGA	998,340 => 998,354	Hypothetical protein	2	1^c^
D	3	GAA	377,718 => 377,732	TIGR02300 family protein	4–6	3 (0.267)

^a^ Letters refer to the TR markers developed by Islam et al. (2012). Other markers were developed in the present study.

^b^ Nei's 1978 gene diversity. Mean *H*_*T*_ = 0.403.

^c^ Polymorphism was observed for samples from other geographic origins (based on blastn queries).

All amplicons produced by simplex PCR from the two reference samples (V01 and V02), as well as rare alleles from field samples, were submitted to Sanger sequencing to check the nucleotide sequence (TRs and flanking regions). PCRs were performed using the BD advantage 2 polymerase mix kit (Takara Bio, Saint‐Germain‐en‐Laye, France). Briefly, 2 μL of 10‐fold‐diluted DNA was used as a template in mixes containing 5 μL of 10× Advantage 2 PCR Buffer 10×, 0.2 mM of dNTPs mix, 0.2 μM of each primer, 1 μL of Advantage 2 polymerase Mix, and RNase‐free water to yield a final volume of 50 μL. PCR amplifications were performed in a Veriti thermocycler as follows: 3 min at 95°C (HotStart activation and DNA initial denaturation), 40 cycles with denaturation at 95°C for 15 s, primer annealing at 64°C for 30 s and elongation at 68°C for 60 s, and then a final extension at 68°C for 7 min. Amplicons were submitted to Sanger sequencing (Genewiz, Takeley, UK) using the same primers as for PCR.

Genetic polymorphism and population structure analyses were only performed with samples having unambiguous MLVA allelic profiles. The Hunter Gaston Discriminatory Index (HGDI) for MLVA‐12 and MLVA‐7 was computed using the DescTools package v.0.99.48 in R (Hunter and Gaston 1988). Stoddart and Taylor's diversity index (*G*) was computed using the rarefaction procedure for unequal sample sizes and the poppr v.2.9.3 package in R (Kamvar, Tabima, and Grünwald 2014; Stoddart and Taylor 1988). Evenness (E5) was computed with the same package. Genetic differentiation (based on *R*_ST_) was computed with the genepop v.1.1.7 package in R (Rousset 2008). Minimum spanning trees were produced using the algorithm recommended for TR data (combining global optimal eBURST [goeBURST] and Euclidean distances) in PHYLOVIZ v.2 (Nascimento et al. 2017) and used to delineate clonal complexes (CC are networks of single‐locus variants [SLVs]), and singletons (haplotypes with no identified SLV). The population structure was analyzed by a discriminant analysis of principal components (DAPCs) using the adegenet v.2.1.7 R package (Jombart 2008; Jombart, Devillard, and Balloux 2010). This method is free of any assumption linked to a population genetic model, which makes it suitable for analyzing datasets of non‐freely recombining organisms. The Réunion dataset had a large number of samples (*n* = 509) and displayed greater genetic diversity than the French West Indies datasets. We examined it for spatial structure using the global .rtest and related functions in the adegenet package in R (Jombart 2008).

### 2.5 Identification of Integrated Prophages

Prophage‐based identification was performed on a subset of 11 and 16 samples originating from the French West Indies (Guadeloupe and Martinique) and Réunion, respectively. The same samples were used for prophage‐ and MITE‐based genotyping (Table S4). The samples were selected based on MLVA profiles in order to cover the genetic diversity highlighted with this technique. Prophage‐based identification targeted four prophage types that were reported in CLas (referred to as T1–T4, hereafter) (Dominguez‐Mirazo, Jin, and Weitz 2019; Zheng et al. 2016, 2018). Seven to nine PCR primer pairs targeting distinct prophage ORFs were used for each prophage type (Table S5). A prophage was considered present when ≥ 75% of the primers yielded PCR amplification (Zheng et al. 2016). The PCR primers used for T1 and T2 prophages were as reported earlier (Zheng et al. 2016), whereas the primers used for T3 and T4 prophage identification were designed using Geneious from template accessions KY661963 (P‐JXGC‐3) and AP014595 (Ishi‐1), respectively (Table S5). The T3 prophage has only been detected in China to date (Zheng et al. 2018). Therefore, one of the primer pairs (GC3‐9) was designed in a DNA helicase gene of JXGC (PJXGC_gp33). It was highly homologous (> 98% identity) to alleles in T1 and T2 prophages (and amplifiable in corresponding samples). It was used as an internal PCR positive control. Similarly, samples not hosting a T2 prophage still produce an amplicon that can be used as a control with the SC2‐8 primer pair (Zheng et al. 2016). Water was used as a negative control.

All PCRs were performed using the Terra PCR Direct Polymerase Mix kit (Takara Bio, Saint‐Germain‐en‐Laye, France). Briefly, 2 μL of 10‐fold‐diluted DNA was used as a template in mixes containing 12.5 μL of 10× Terra PCR direct buffer, 2.5 μL of 5× Q‐solution (Qiagen), 0.3 μM of each primer (Table S5), 0.625 U of Terra PCR Direct polymerase Mix, and RNase‐free water to yield a final volume of 25 μL. PCR amplifications were performed in a Veriti thermocycler under the following conditions: 2 min at 98°C (HotStart activation), 40 cycles consisting of 98°C for 10 s, annealing temperature for 15 s, extension at 72°C for 90 s, and a final extension at 72°C for 5 min. PCR products were resolved on a Qiaxcel advanced system (Qiagen, Courtaboeuf, France) or slab gel electrophoresis (2% agarose).

### 2.6 Genetic Relatedness Among CLas Strains Based on MITE Sequences

MITE sequences were produced from the same samples as for prophage‐based identification typing (Table S4). PCR was performed using the BD advantage 2 polymerase mix kit (Takara Bio, Saint‐Germain‐en‐Laye, France). Briefly, 2 μL of 10‐fold‐diluted DNA was used as a template in mixes containing 5 μL of 10× Advantage 2 PCR Buffer SA 10×, 0.2 mM of dNTPs mix, 0.2 μM of each primer (LapPF1‐f GCCACTTTGGGGTAGCAGTA, LapPF1‐r AAAACTTTCGTCACGGCTTT) (Wang et al. 2013), 1 μL of Advantage 2 polymerase mix, and RNase‐free water to yield a final volume of 50 μL. PCR amplifications were performed in a Veriti thermocycler under the conditions recommended by the manufacturer: primer annealing temperatures at 72°C for samples from Réunion Island and 70°C for samples from Guadeloupe and Martinique for 30 s, extension at 68°C for 1 min and then a final extension at 68°C for 7 min. PCR products were resolved on a Qiaxcel advanced system (Qiagen, Courtaboeuf, France) or slab gel electrophoresis (2% agarose). When two amplicons were detected, they were separated from each other and purified from agarose gel using Illustra GFX PCR DNA. Samples for which single amplicons were produced, they were purified using 20 μL of PCR product and 0.6× of Sera‐Mag Select magnetic beads (Cytiva, Villacoublay, France), as recommended by the manufacturer. Once amplicons were purified, they were sequenced using the BigDye Terminator v.3.1 Cycle Sequencing Kit (Applied Biosystems, Courtaboeuf, France): 5–20 ng of PCR product was used as a template in mixtures containing 2 μL of BigDye Terminator V3.1, 1 μL of 5X sequencing buffer, 1 μL of primer at 5 μM, and water to yield a final volume of 10 μL. Sequencing amplifications were performed in a Veriti thermal cycler under the conditions recommended by the manufacturer. Sequencing products were then purified using the BigDye XTerminator (Applied Biosystems Courtaboeuf, France), as recommended by the manufacturer. Capillary electrophoresis was performed in an ABI PRISM 3500XL genetic analyzer (Applied Biosystems, Courtaboeuf, France) with a 50 cm capillary array.

Miniature inverted‐repeat transposable elements sequences produced from Réunion and the French West Indies samples were compared to reference sequences obtained from Genbank (Wang et al. 2013; Table S4) or mined as follows: (1) FastQ files for all citrus, ACP, and AfCP SRA accessions were obtained on April 5, 2023 from the NCBI SRA repository using prefetch v.2.11.3 and fastq‐dump from the SRA toolkit (https://github.com/ncbi/sra‐tools); (2) reads were blasted against CLas MITE sequence using blastn v.2.12.0+ (Camacho et al. 2009); (3) reads with a percentage of identity > 70% were mapped to the MITE sequence using bwa v.0.7.17‐r1188 (Li and Durbin 2009); and (4) consensus sequences were built using samtools v.1.13 and bcftools v.1.13 (Danecek et al. 2021; Li et al. 2009). A bootstrapped tree (*n* = 1000) rooted on the most divergent sequences (Wang et al. 2013) was built in Geneious using the neighbor‐joining method and the Jukes–Cantor model. Nucleotide positions where gaps were recorded in the alignment were not considered for building the neighbor‐joining tree.

### 2.7 Phylogenomic Analysis of CLas

The genome sequences of 53 CLas isolates from 11 countries, including one from Réunion and 30 from the United States, were downloaded from the NCBI assembly database or from the figshare repository (https://doi.org/10.6084/m9.figshare.c.5810090.v1) and aligned with the reference CLas strain GXPSY genome (CP004005) using Minimap2 v.2.24 (Table S6). The aligned reads were sorted using Samtools v.1.13 and single‐nucleotide variations (SNVs) were called using BCFtools v.1.13 with the haploid model. We used VCFtools v.0.1.16 (Danecek et al. 2011) to filter any genomes with a high proportion of missing genotypes (above 30%), and any variant with more than 20% of missing genotypes and a minor allele frequency lower than or equal to 1%. After filtering, the remaining dataset included a total of 52 genomes and a total of 2134 bi‐allelic SNPs. We created consensus FASTA sequences using vcf2fasta_consensus.py (https://github.com/stsmall/An_funestus/tree/master/vcf/). We reconstructed the phylogenetic relationships between genomes using the ML approach implemented in RAxML‐NG v.1.1.0 with a GTR + G substitution model. We assessed the node's support using 1000 bootstrap replicates (Kozlov et al. 2019). The resulting phylogenetic trees were visualized using FigTree v.1.4.4 (https://github.com/rambaut/figtree/releases). The detection of recombinant sequences within the core genome alignment was performed using ClonalFrameML v.1.12 with the ML tree produced by RAxML‐NG as the starting tree (Didelot and Wilson 2015). The recombinant events detected were discarded from the SNP matrix using a custom python script (ExclRecPos.py) and VCFtools. None of the bi‐allelic SNPs were located in the recombinant events detected, which confirms that only SNPs caused by mutations were used to reconstruct the phylogenetic relationships.

## 3 Results

### 3.1 Preliminary Characterization and Identification at Species Level

Nearly all (99%) of the gDNA samples from set (i) (see section 2.1), initially tested CLas‐positive by endpoint PCR, were confirmed as positive by real‐time PCR in our facility. In Réunion, 19% and 18% of samples collected from single trees in commercial groves, that is, set (ii), were scored as markedly positive (Cq ≤ 30) or weakly positive (30 < Cq < 36), respectively. These rates were 21% and 33%, respectively, for backyard trees, that is, set (iii). Among the positive samples collected at high elevations (≥ 800 masl; *n* = 115), only three were shown to contain high titers of *Ca*. Liberibacter (asiaticus Cq ≈ 22; africanus Cq ≈ 24). All other samples that were found weakly positive for both the CLas and the CLaf real‐time PCR assays showed slightly delayed Cq for CLaf. The Cq values were almost always delayed by approximately 0.5–3 Cq (or undetermined) for the latter assay. A subset of samples (*n* = 40) was assayed with Osman's real‐time PCR assays but did not show any clear improvement in terms of analytical specificity. The sole noticeable exception was that of four samples from a single site. They were scored as markedly positive using the real‐time PCR assays developed by Li, Hartung, and Levy (2006) and showed a slightly delayed response for CLas.

**FIGURE 2:**
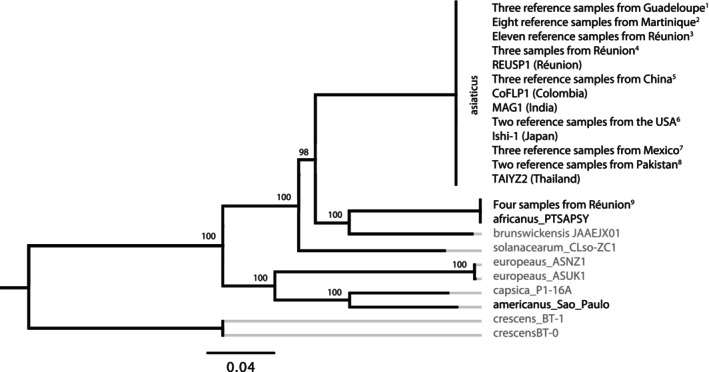
Maximum‐likelihood (ML) tree derived from the GTR + G evolution model, based on a concatenated dataset (total length 3523 bp) of seven housekeeping gene portions (*adk*, *atpA*, *fbpA*, *ftsZ*, *glyA*, *groEL*, and *gyrB*), showing the relationships between species of *Liberibacter* and “*Candidatus* Liberibacter”. All field samples sequenced herein shared 100% identity with reference sequences of “*Ca*. Liberibacter africanus” (CLaf) or “*Ca*. Liberibacter asiaticus” (CLas). Support is shown for bootstrap values greater than 80%. Citrus pathogens are shown as black labels. Additional information on samples and label codes is provided in Table S1.

Most samples from Réunion and all samples from the French West Indies that were assayed by MLSA were confirmed as CLas, showing 100% sequence identity with CLas sequences from public databases (Figure 2). This technique also clearly identified four samples as CLaf (99.97% sequence identity to CLaf PTSAPSY; one SNP on *ftsZ*; total sequence length 4188 bp) (Figure 2). These four samples originated from a single site in Réunion (HLB20‐017, HLB20‐018, HLB20‐019, and HLB20‐020), confirming CLaf presence after three decades during which it was not reported.

### 3.2 CLas Genetic Polymorphism and Population Structure From TR Data

Tandem repeat‐based genotyping with MLVA‐12 yielded amplicons at all targeted loci for all samples originating from the French West Indies. Out of the 513 samples from Réunion, only the four samples identified as CLaf by MLSA repeatedly produced no amplicon, whatever the targeted locus. MLVA‐12 identified 182, 38, and 37 haplotypes among 509, 148, and 129 samples from Réunion, Guadeloupe, and Martinique, respectively. All TR markers, except G, were polymorphic in our dataset and yielded three to 18 alleles (Table 1). Sanger sequencing of TRs confirmed rarely detected allelic states, as well as those that differed by ≥ 3 repeats from their closest sized relative. The discriminatory power of the MLVA‐12 scheme was nearly maximal (HGDI = 0.990). As expected, it exceeded that of MLVA‐7 (HGDI = 0.969; 99, 16, and 10 haplotypes detected in Réunion, Guadeloupe, and Martinique, respectively).

At the island scale, rarefied Stoddart and Taylor's diversity index was significantly larger in Réunion (*G* = 47.63; 95% confidence interval 37.40–58.80) compared to the two Caribbean islands (Guadeloupe *G* = 10.15; 95% confidence interval 9.09–11.44 and Martinique *G* = 14.16). Consistently, 34, five, and zero private alleles (i.e., alleles only present in samples from a single island) were identified in Réunion, Guadeloupe, and Martinique, respectively. The datasets rarely included frequent haplotypes and consistently yielded quite high evenness values (E5 ≈ 0.6).

**FIGURE 3:**
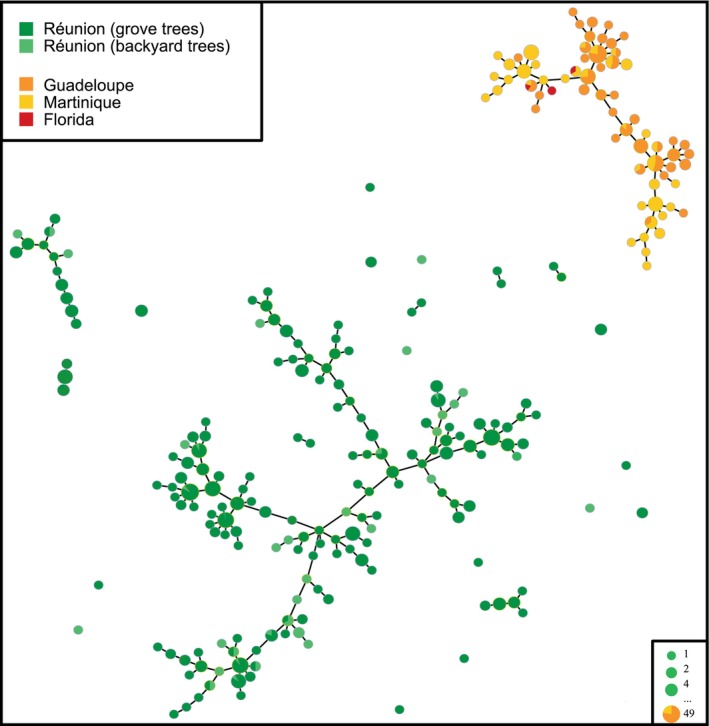
Minimum spanning tree from MLVA‐12 data showing the genetic structure of “*Candidatus* Liberibacter asiaticus” (CLas) in three French outermost regions of the European Union. Dots represent haplotypes. Dot diameter and color represent the number of samples per haplotype and geographic origin, respectively. All distinct networks are clonal complexes (see the 2. Materials and Methods section for more details). As our Chinese reference samples (see text for details) were not informative, that is, not closely related to any of our samples, they were not included in the figure.

Unlike MLVA‐7, MLVA‐12 made it possible to distinguish between haplotypes from Réunion and the French West Indies (Figure 3). A highly significant *R*_ST_‐based genetic differentiation was found between populations in Réunion and the French West Indies (Guadeloupe: 0.568 and Martinique: 0.487). In contrast, many MLVA‐12 haplotypes were shared between samples from Guadeloupe and Martinique, yielding a lower but significant genetic differentiation (*R*_ST_ = 0.276). Reference samples from Florida (i.e., samples used to confirm consistency with previously published data, Islam et al. 2012) either shared identical haplotypes with Caribbean samples or were SLVs (Figure 3). When the datasets from the two Caribbean islands were merged, all samples clustered into a single CC. In contrast, the minimum spanning tree built from Réunion samples splits haplotypes into 22 distinct CCs or singletons that were delineated by double‐locus variations (Figure 3). Consistent with this absence of distantly related clusters in the Réunion dataset, the DAPC *k*‐means analysis suggested an absence of clear genetic structure (Figure S1).

Although we did not find a global spatial structure (*p* = 0.709) in the Réunion dataset, we revealed a weak but significant local structure (*p* = 0.037), consistent with frequent occurrences of genetically different samples collected from spatially close trees. No significant genetic differentiation was found between samples from grove and backyard trees (*R*_ST_ = 0.009), consistent with the structure of the haplotype network produced (Figure 3) and multiple occurrences of identical haplotypes detected from backyard trees and commercial grove trees located in the vicinity. Rarefied G values estimated for each local population sampled in Réunion (samples collected from at least eight distinct sites in a single locality and totalling ≥ 30 samples) did not differ significantly. Weak but significant genetic differentiation (0.099 ≤ *R*_ST_ ≤ 0.258) was recorded among local populations.

The sampling design allowed us to detect polymorphism at a tree scale. It was detected in 24% of the sampled trees from Réunion. Most cases involved single‐locus variations at a TR locus displaying a high genetic diversity, primarily A and E, and also C, T03, and T09 (Table 1). It is interesting to note that 6% of the sampled trees displayed polymorphism at three to six TR loci, which suggests that co‐infections may have occurred.

### 3.3 Identification of Integrated Prophages

PCR data indicated that all CLas samples from Réunion hosted T1 and T4 prophages. The primer pairs, SC2‐8 for T2 (Zheng et al. 2016) and GC3‐9 for T3 (Zheng et al. 2018), yielded the expected control amplicon for both prophages in all samples, except the negative control. Similar to samples from Brazil, a 1.3 kb amplicon was detected from all Réunion samples from PCR performed with the SC1‐7 primer pair (DaSilva et al. 2019). Samples originating from the French West Indies differed from samples from Réunion, as they all hosted T1, T2, and T4 prophages. When analyzed with the SC2‐1 primer pair, samples from the French West Indies yielded two amplicons, one of which was of the expected size. The SC1‐7 amplicon was of the same size as of the samples previously reported from China (Zheng et al. 2016) but differed from the Réunion samples (0.9 vs. 1.3 kb). We did not observe any within‐island variation in prophage amplicon profiles.

### 3.4 Genetic Relatedness Among CLas Strains Based on MITE Sequences

**FIGURE 4:**
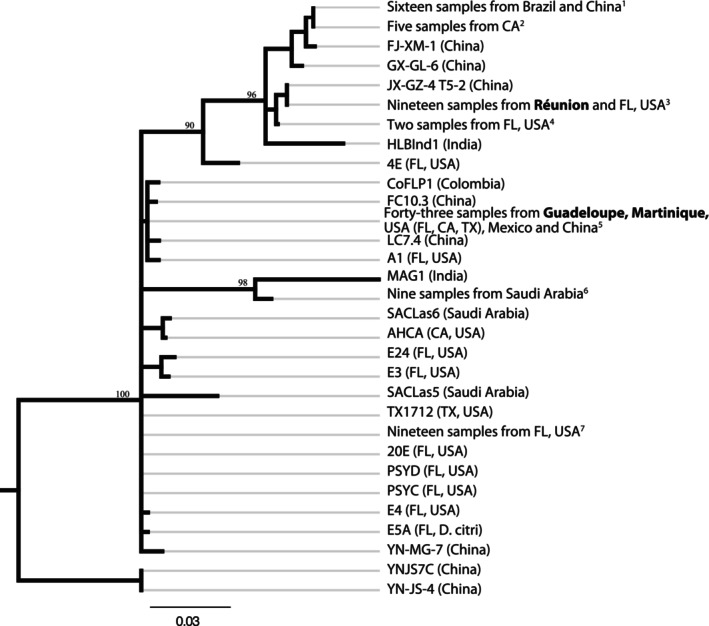
Neighbor‐joining tree, based on the MCLas‐B miniature inverted‐repeat transposable element (MITE) nucleotide sequence, showing the relationships between “*Candidatus* Liberibacter asiaticus” (CLas) samples originating from three EU outermost regions and samples from several continents where HLB has become established. Support is shown for bootstrap values greater than 80%. Additional information on samples and label codes is provided in Table S4.

Consistent with its low polymorphism (Wang et al. 2013), the MCLas‐A element that was detected in all samples from Guadeloupe and Martinique displayed no sequence polymorphism. This element was absent in Réunion samples, which is fully consistent with prophage fingerprints. The sequence of this element was 100% identical to that in the SC2 prophage (Zhang et al. 2011) and in most samples obtained from Genbank (Wang et al. 2013). The MCLas‐B element, which was previously found most useful for MITE typing (Wang et al. 2013), was subject to further analysis. All sequences produced herein had the typical structure as that described in previous publications, that is, TA direct repeats and 22–23 bp inverted repeats (IRs) flanking the element (Wang et al. 2013). A total of 31 sequence types (STs) were identified in the dataset (Figure 4). The MCLas‐B element displayed no sequence polymorphism among samples originating from the same EU outermost region. All samples from Guadeloupe and Martinique shared the same ST. This ST was 100% identical to that in the SC1 prophage (Zhang et al. 2011) and in several samples from China, three US states (California, Florida, and Texas), and Mexico (Figure 4). Samples from (i) the French West Indies and (ii) Réunion were assigned to two distinct STs that differed by 25 SNPs. The Réunion ST was 100% identical to three accessions from Florida (SRR16895070, SRR16895076, and SRR16895081) and highly similar to that of a Chinese sample collected in the Jiangxi province (KP338037). The latter only differed by a 3 bp insertion in its left IR sequence.

### 3.5 Phylogenomic Relationships of CLas

To explore the potential source(s) of the population(s) responsible for the CLas outbreak in Réunion, we reconstructed a ML phylogeny using 52 CLas whole genomes, including one CLas genome from Réunion (ReuSP1; Lu et al. 2021), and a total of 2134 non‐recombinant bi‐allelic SNPs (Figure S2 and Table S6). While the phylogenomic tree was consistent with previous studies (Gao et al. 2022; Higgins, Mann, and Heck 2022), ReuSP1 did not cluster with any other CLas clade, that is, did not directly descended from one of the sequenced CLas strains. As genomic resources were not available for the CLas strains from Martinique and Guadeloupe, we could not use this complementary approach to support our genetic marker‐based data.

## 4 Discussion

In the present study, we genetically characterized emerging populations of *Candidatus* Liberibacter responsible for HLB in three outermost regions of the EU using several marker‐based genotyping techniques. HLB emerged in the 2010s in all three territories, either as first reports (French West Indies) or as a new alert after two decades with no reports of the disease (Réunion), which followed a massive outbreak that occurred from the late 1960s until the early 1990s (Aubert, Bové, and Etienne 1980; Bové 2006; Cellier et al. 2014).

### 4.1 The Putative Source(s) of *Candidatus* Liberibacter in the French West Indies

The movement of citrus material from Réunion to Martinique in the 2010s is likely to be at the origin of the establishment of another citrus bacterial pathogen, *Xanthomonas citri* pv. *citri* (the causal agent of Asiatic canker), in Martinique (Richard et al. 2016, 2021). Herein, our genotyping data targeted several types of markers and invalidated the hypothesis that this citrus material could have subsequently disseminated CLas. The samples from Réunion and Martinique were clearly distinguished using TR‐based typing. They also differed in terms of prophage and MITE content. The populations that concomitantly emerged in Guadeloupe and Martinique were grouped into a single CC with no apparent structure, using TR‐based markers, and shared otherwise identical MITE ST and prophage fingerprints. Their close relatedness suggests that there was a single introduction of the pathogen in the French West Indies or multiple introductions of highly similar strains, and possible exchanges between the two islands. Our MLVA‐12 data also suggested a close relatedness between the populations associated with HLB emergence in the French West Indies and some strains from Florida. MITE typing confirmed the genetic relatedness between French West Indies samples and some accessions from Florida, in addition to two other US states (California and Texas) and two other countries (China and Mexico). Additional WGS data will be required to further our understanding of the source(s) of CLas in the French West Indies.

### 4.2 HLB Reemergence in Réunion

Consistent with French official surveillance data, our results suggested that the ongoing outbreak in Réunion is mainly caused by CLas. The current official real‐time PCR diagnostics method has specificity issues. Therefore, we developed new primers designed for CLaf and CLas, which targeted seven housekeeping gene portions previously defined for phylogenetic analyses of *Ca*. Liberibacter solanacearum (Haapalainen et al. 2018). This MLSA approach overcame the limitations of single‐gene sequencing (Gevers et al. 2005) and allowed the unambiguous identification of all critical samples of CLaf and CLas. Surprisingly, we identified CLaf at very low rates, four out of 509 samples with low Cq (≤ 30) values, from a single locality in Réunion, despite the fact that it has not been recorded on the island for three decades. However, the exact distribution of CLaf in Réunion remains unknown, as our molecular analysis procedure did not allow to decipher mixed infections nor to identify the *Ca*. Liberibacter species in high‐Cq samples.

The amount of genetic diversity revealed by TR markers among CLas samples from Réunion largely exceeded that estimated from samples originating from Guadeloupe and Martinique, although the disease was recorded almost concomitantly in 2012, 2013, and 2015 in Guadeloupe, Martinique, and Réunion, respectively. Evenness was around 0.6 in all three outermost regions, which suggests the absence of epidemic clonality and the pathogen's moderately active transmission. Despite the massive disease management campaign conducted in Réunion in the 1970s and 1980s, HLB was still present on the island at very low rates in the mid‐1990s (Aubert et al. 1996). The disease appears to have been overlooked before being officially reidentified in 2015. This is supported by the relatively frequent occurrence of multiallelic profiles at ≥ 3 TR loci from gDNA obtained from a single midrib. Our finding suggests that time between first infection and sampling was sufficient to allow multiple infections or diversification of allelic states at multiple loci. This result is also consistent with (i) Google satellite image history of the area where the 2015 official identification took place and where major tree decline in the grove appears to have occurred years earlier, and (ii) the fast and concomitant detection of CLas from a fairly high number of Réunion localities, after the official detection of the disease was announced in 2015. Similar situations have occurred in other cases of HLB emergence, for example, in (i) Sao Paulo State (Brazil) where some data suggest that the pathogen may have been introduced approximately 10 years prior to detection and (ii) Florida where the initial discovery reported in 2005 prompted an extensive survey that revealed remote HLB foci in commercial groves and residential areas that were more than 100 km away from the primary focus in some cases (Gottwald 2010).

Herein, we revealed the absence of genetic differentiation between samples originating from grove trees versus backyard trees and multiple occurrences of identical haplotypes on backyard trees and grove trees located in the vicinity. Frequent HLB occurrences were found on backyard trees. This finding suggests that they may constitute a disease reservoir, by relaying psyllid spread and, thus, contributing to disease progress. The discriminatory power of MLVA‐12 makes it a useful tool for evaluating the genetic relatedness between samples originating from isolated trees and commercial groves. It will provide a valuable contribution to quantitative epidemiology data in future studies addressing this issue (Pazolini et al. 2021).

Our study shows that the altitudinal distribution of CLas in Réunion (0–950 masl) is more extensive than suggested by previous assessments (Aubert 1987). Indeed, accurate diagnostic techniques were not available at the time of the first major HLB outbreak. Consequently, the altitudinal distribution of ACP, using morphological estimators for vector identification, was used as a proxy. However, a recent study suggests that this estimation was probably incorrect because CLas was effectively transmitted by AfCP (Reynaud et al. 2022), which was commonly detected in the highlands in the 1970s (Aubert, Bové, and Etienne 1980). Several factors could probably explain these observations at high elevation. However, one explanation may be partly related to the availability of more sensitive molecular detection techniques, as Cq values from real‐time PCR indicated that the vast majority of samples collected at elevations ≥ 800 masl were found to host low CLas titers. Low temperatures at these elevations may restrict the multiplication and within‐plant movement of CLas. This finding is in line with previous studies, which (i) suggested through modeling the negative impact of low temperatures on CLas (Narouei‐Khandan et al. 2016) and (ii) underlined the lack of CLas colonization in graft‐inoculated plants subjected to a temperature range of 8°C–20°C (Raiol‐Junior et al. 2021). Weather data mining indicated that the mean daily number of hours, with temperatures ≤ 20°C at sampled sites located at elevations ≥ 800 masl, ranged between 15 and 20 over the sampling period.

Our results revealed a lack of global spatial structure in Réunion, which suggests that ample movements of strains have occurred repeatedly across the island since the first outbreaks. All human‐mediated pathways conducive to the movement of CLas between groves reported to date, including the movement of citrus budwood or nursery plants, which are contaminated or bear contaminated *D. citri* , and transport of unprocessed fruit in trailers may be involved in Réunion (Gottwald, Graça, and Bassanezi 2007; Halbert et al. 2010, 2012). However, the relative importance of these different pathways of transmission remains unknown. One case of an HLB‐positive nursery consignment has been detected in the timeframe of the present study. Our results prompted the implementation in 2022 of official regulations (including the compulsory production of citrus nursery plants in insect‐proof screenhouses from HLB‐indexed budwood) by the local regulatory agency. In addition to human‐mediated long‐distance dissemination, medium‐ to long‐distance dissemination of CLas through the active or passive movement of infected psyllids has been suggested. This pathway for the dispersal of HLB vectors and Ca. *Liberibacter* spp. has not been studied in depth. More data are clearly required to document this (Gottwald 2010). The issue was recently brought to light in relation to malaria, a major disease caused by mosquito‐transmitted *Plasmodium* that is responsible for hundreds of thousands of human casualties every year, particularly in Africa, which pays a high price. The mystery behind the population boom of malaria vectors in semi‐desert areas after extended, very dry periods was solved by high‐altitude air sampling and modelling. These techniques provided evidence of the seasonal long‐distance movement of large numbers of insects, which follow wind flows, and identified six species of malaria vectors (Besansky 2019; Huestis et al. 2019).

Interestingly, MLVA‐12 data from Réunion did not support the co‐occurrence of distinct CLas clades resulting from multiple independent introductions. However, we cannot exclude that multiple introductions of genetically related bacteria did occur. This contrasts with a previous study based on mitochondrial cytochrome oxidase I sequences of a worldwide collection of *D. citri* , which suggested that two distinct psyllid variants were present in Réunion (Boykin et al. 2012). MITE typing suggested a close genetic relatedness of Réunion CLas strains with strains from Florida (USA) and Jangxi (China). We attempted to use the only recently released whole‐genome sequence of a CLas sample from Réunion (ReuSP1; Lu et al. 2021) but did not find any closely related sequences among the publicly available genomes (Table S6). More CLas genomic resources from Réunion will thus be required to identify the source of the population responsible for the CLas outbreak on the island.

### 4.3 Global Perspectives for HLB Surveillance

HLB represents a severe threat to citrus industries globally. Available studies underline the need to improve surveillance and implement responsive outbreak investigations of HLB‐causing *Ca*. Liberibacter, which are among the top 20 priority pests on the EU list. Improving surveillance requires (i) robust diagnostic tools, (ii) powerful and high‐throughput strain tracing involving marker‐based genotyping (primarily for small‐scale outbreak investigations) and evolutionary genomics, and (iii) new analytical surveillance approaches. These include modelling strategies involving data networks linked to climate change, agricultural landscapes, and abiotic factors, and new surveillance indicators or strategies, such as text mining, aerial surveillance of insect vectors in crucial areas, and next‐generation biomonitoring (Choufany et al. 2021; Derozier et al. 2023; Eck et al. 2022; Makiola et al. 2020; Morris et al. 2022; Richard et al. 2023).

Consistent with a previous study conducted in East Africa (Roberts et al. 2017), we showed that a widely used real‐time PCR assay (Li, Hartung, and Levy 2006) and a recent alternative (Osman et al. 2023) failed to robustly differentiate between CLaf and CLas. Other new diagnostic techniques based on loop‐mediated isothermal amplification (LAMP) or recombinase polymerase amplification (RPA) have been developed for CLas (Keremane et al. 2015; Li, Hartung, and Levy 2006; Morán et al. 2023). However, they are not yet available for CLaf (DaGraca et al. 2022). Similarly, only WGS data can provide sufficiently high resolution to robustly evaluate the phylogenomic relatedness between CLas strains. A breakthrough in (i) the availability of robust diagnostics and strain tracing tools for surveillance, and (ii) the understanding of the epidemiology, ecology, and evolutionary history of HLB‐causing *Ca*. Liberibacter will require a massive international sequencing effort. Whole‐genome assemblies will provide a valuable resource for reconstructing the probable routes and the timing of invasions. This will also improve our understanding of the molecular basis of virulence and shed light on the host–pathogen–vector interactions and, thereby, contribute to the development of more effective disease control strategies that target *Ca*. Liberibacter. Infected citrus herbarium collections could represent another important source of dated and preserved DNA, which could, together with modern genomes, shed light on the evolutionary history of HLB‐causing *Ca*. Liberibacter species.

## Conflicts of Interest

The authors declare no conflicts of interest.

## Supporting information


**Table S2.** Primers used and annealing temperatures for amplification and sequencing of CLaf and CLas in the MLSA scheme.
**Table S3.** Primers and PCR conditions used to amplify CLas TR markers in the MLVA‐12 scheme.
**Table S4.** Information on the *Candidatus* Liberibacter asiaticus (CLas) samples used in the MITE MCLas‐B analysis.
**Table S5.** Primers used and annealing temperatures for amplification of CLas prophage regions.
**Table S6.** Information on the CLas genomes used in this study.

**Figure S1.**Bayesian information criterion derived from the DAPC k‐means analysis performed on “*Candidatus* Liberibacter asiaticus” (CLas) tandem‐repeat (TR) data sampled in Réunion.

**Figure S2.**Maximum‐likelihood phylogenetic tree of the “*Candidatus* Liberibacter asiaticus” (CLas) whole‐genome from public databases. Support is shown for bootstrap values greater than 80%. The recently released whole‐genome sequence of a CLas sample from Réunion (Lu et al. 2021) is highlighted in bold.

## Data Availability

DNA sequence data have been deposited in GenBank, https://www.ncbi.nlm.nih.gov/genbank. The microsatellite data that support the findings of this study are available in the CIRAD Dataverse at https://dataverse.cirad.fr/dataverse/pvbmt. The custom python scripts used in this study, including the script to automatically download GenBank assemblies for a given search term, are available at GitHub (https://github.com/fredericlabbe/CLas_Phylogenomics.com/fredericlabbe/CLas_Phylogenomics).
