# Supplementary figures and images for: Genetic Signatures of Contrasted Outbreak Histories of “Candidatus Liberibacter asiaticus”, the Bacterium That Causes Citrus Huanglongbing, in Three Outermost Regions of the European Union
Source: Evol Appl. 2024 Dec 16;17(12):e70053. doi: 10.1111/eva.70053 (PMC11649586; doi:10.1111/eva.70053)

Fig. S1

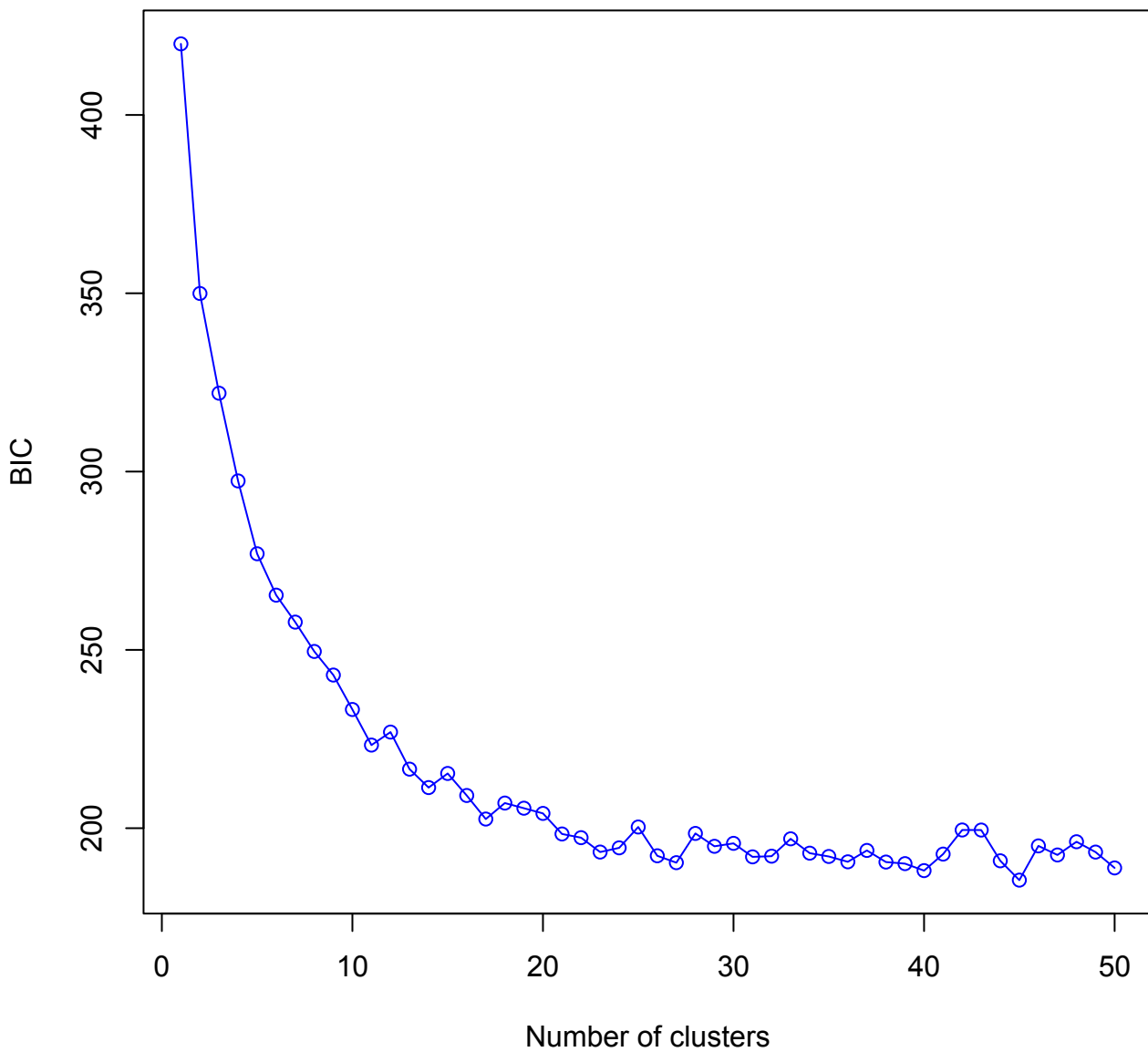

Supplement: Supplementary file 2 — Figure S1.Bayesian information criterion derived from the DAPC k‐means analysis performed on “Candidatus Liberibacter asiaticus” (CLas) tandem‐repeat (TR) data sampled in Réunion. [file EVA-17-e70053-s003.pdf]

Fig. S2

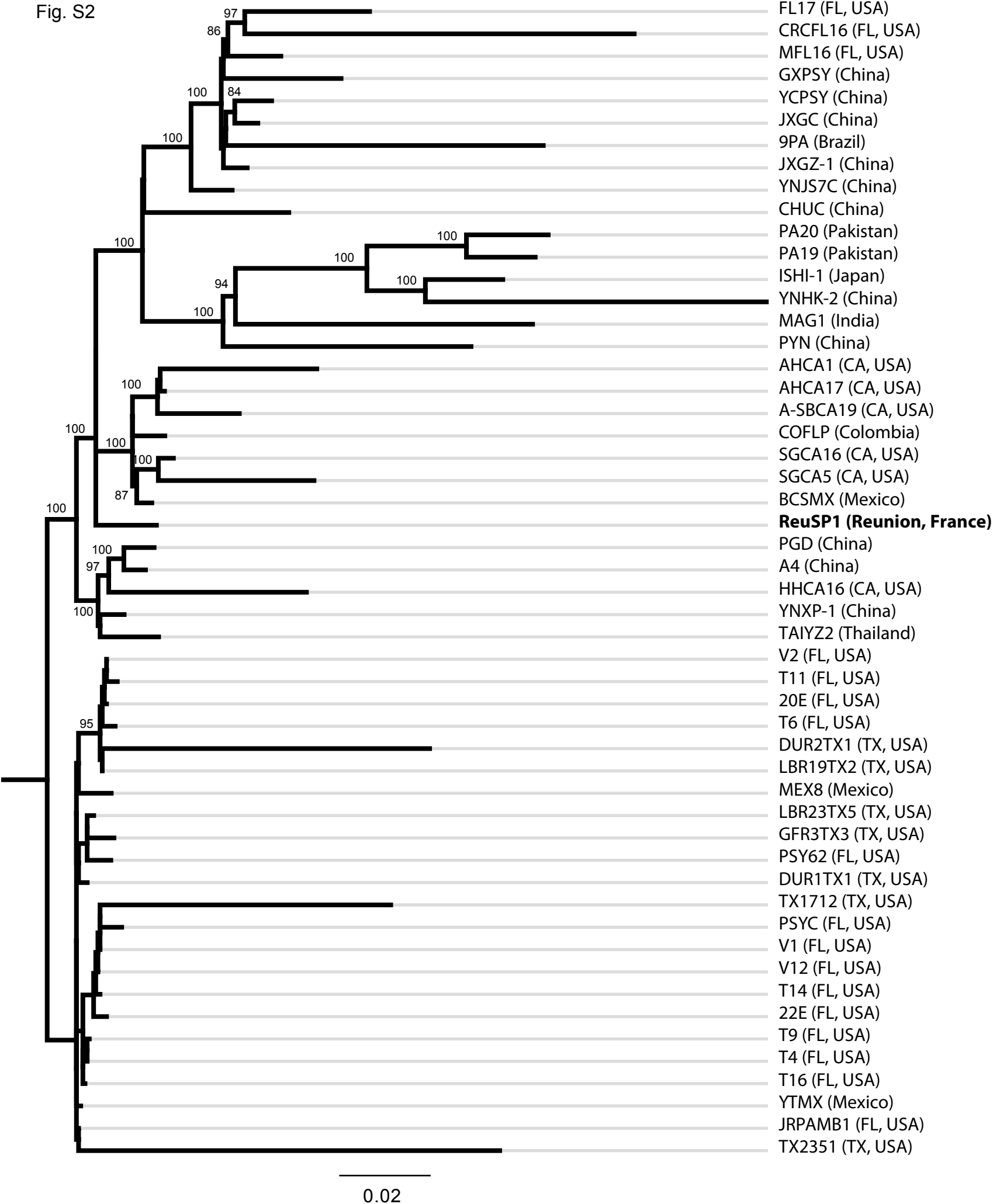

Supplement: Supplementary file 3 — Figure S2.Maximum‐likelihood phylogenetic tree of the “Candidatus Liberibacter asiaticus” (CLas) whole‐genome from public databases. Support is shown for bootstrap values greater than 80%. The recently released whole‐genome sequence of a CLas sample from Réunion (Lu et al. 2021) is highlighted in bold. [file EVA-17-e70053-s002.pdf]
